# Breast cancer cell-derived extracellular vesicles transfer miR-182-5p and promote breast carcinogenesis via the CMTM7/EGFR/AKT axis

**DOI:** 10.1186/s10020-021-00338-8

**Published:** 2021-07-16

**Authors:** Chong Lu, Yu Zhao, Jing Wang, Wei Shi, Fang Dong, Yue Xin, Xiangwang Zhao, Chunping Liu

**Affiliations:** Department of breast and thyroid surgery, Union Hospital, Tongji Medical College, Huazhong University of Science and Technology, 1277 Jiefang Avenue, Wuhan, 430022 Hubei China

**Keywords:** Breast cancer, Extracellular vesicles, miR-182-5p, CMTM7, EGFR/AKT signaling pathway, Angiogenesis, Human umbilical vein endothelial cells

## Abstract

**Background:**

Extracellular vesicles (EVs) derived from tumor cells are implicated in the progression of malignancies through the transfer of molecular cargo microRNAs (miRNAs or miRs). We aimed to explore the role of EVs derived from breast cancer cells carrying miR-182-5p in the occurrence and development of breast cancer.

**Methods:**

Differentially expressed miRNAs and their downstream target genes related to breast cancer were screened through GEO and TCGA databases. miR-182-5p expression was examined in cancer tissues and adjacent normal tissues from patients with breast cancer. EVs were isolated from breast cancer cell line MDA-MB-231 cells and identified. The gain- and loss-of function approaches of miR-182-5p and CKLF-like MARVEL transmembrane domain-containing 7 (CMTM7) were performed in MDA-MB-231 cells and the isolated EVs. Human umbilical vein endothelial cells (HUVECs) were subjected to co-culture with MDA-MB-231 cell-derived EVs and biological behaviors were detected by CCK-8 assay, flow cytometry, immunohistochemical staining, Transwell assay and vessel-like tube formation in vitro. A xenograft mouse model in nude mice was established to observe the tumorigenesis and metastasis of breast cancer cells in vivo.

**Results:**

miR-182-5p was highly expressed in breast cancer tissues and cells, and this high expression was associated with poor prognosis of breast cancer patients. miR-182-5p overexpression was shown to promote tumor angiogenesis in breast cancer. Moreover, our data indicated that miR-182-5p was highly enriched in EVs from MDA-MD-231 cells and then ultimately enhanced the proliferation, migration, and angiogenesis of HUVECs in vitro and in vivo. Moreover, we found that CMTM7 is a target of miR-182-5p. EVs-miR-182-5p promotes tumorigenesis and metastasis of breast cancer cells by regulating the CMTM7/EGFR/AKT signaling axis.

**Conclusions:**

Taken altogether, our findings demonstrates that EVs secreted by breast cancer cells could carry miR-182-5p to aggravate breast cancer through downregulating CMTM7 expression and activating the EGFR/AKT signaling pathway.

**Supplementary Information:**

The online version contains supplementary material available at 10.1186/s10020-021-00338-8.

## Introduction

Breast cancer is the most frequently diagnosed cancer worldwide (Harbeck and Gnant 2017) and represents a leading cause of premature mortality among women, risk factors for which include race, ethnicity, family history and genetic traits, along with excess alcohol consumption, physical inactivity and exogenous hormones (Coughlin 2019). Despite the progress made in the screening, diagnosis and therapeutic strategies in breast cancer, poor prognosis and drug resistance present major challenges, as well as the rising incidence and mortality rates in the developing parts (Fahad 2019). Finding a possible molecular target would be of clinical significance to improve the survival and safety outcomes for patients with breast cancer.

Extracellular vesicles (EVs) are membrane vesicles that are released by most cells into the surrounding extracellular environment and can be divided into different subgroups: apoptotic bodies, microvesicles, and exosomes (Raposo and Stoorvogel 2013). Tumor cell-derived EVs hold great potential for clinical application in cancer early diagnosis, prognosis, and treatment response due to their properties to transferring the one of bioactive cargoes microRNAs (miRNAs) to recipient cells (Rahbarghazi 2019). miRNAs, a class of non-coding RNA approximately 22 nt in length, exert crucial roles in the initiation and progression of cancer, acting either as oncomiRs or as tumor suppressors through several molecular mechanisms (Markopoulos 2017). miR-182 inhibition contributes to suppressed epithelial-mesenchymal transition, invasion and migration of breast cancer cells in vitro along with tumor growth in vivo (Mao 2020). TargetScan website predicted the binding sites between miR-182-5p and CKLF-like MARVEL transmembrane domain-containing 7 (CMTM7), a member of the CMTM family playing key roles in the occurrence and progression of cancer. CMTM7 has been reported to be down-regulated in gastric cancer and this downregulation facilitates the proliferation and tumorigenesis of gastric cancer cells in vitro and in vivo (Jin et al. 2018). In addition, a previous study showed that knockdown of CMTM7 promotes the malignant potential of non-small cell lung cancer cells and activates the epidermal growth factor receptor/protein kinase B (EGFR/AKT) signaling pathway by decreasing EGFR internalization and degradation (Liu 2015). Importantly, the inhibited EGFR/AKT signaling pathway is responsible for the anti-proliferative and anti-migration activities of actein on breast cancer cells (Wu 2018). On the basis of above literature and findings, we proposed the hypothesis that breast cancer cell derived-encapsulated miR-182-5p regulates the CMTM7/EGFR/AKT signaling pathway, thus contributing to the development and progression of breast cancer. Hence, the current study aims to validate if the aforementioned hypothesis was valid and to further explore to further explore the mechanisms by which miR-182-5p affects the HUVECs proliferation, migration, and angiogenesis through regulation of CMTM7 expression.

## Materials and methods

### Ethics statement

The current study was approved by the Ethics Committee of our hospital and performed in strict accordance with the Declaration of Helsinki. All participants signed informed consent documentation before sample collection. Animal experiments were approved by the Animal Ethics Committee of our college and strictly performed according to the Guide for the Care and Use of Laboratory Animals published by the US National Institutes of Health. Extensive efforts were made to ensure minimal suffering of the included animals.

### Microarray-based gene expression profiling

Through the R language "limma" package (http://www.bioconductor.org/packages/release/bioc/html/limma.html), differential analysis was performed on the breast cancer miRNA expression datasets GSE58027, GSE45666, GSE35412-1, GSE35412-2 and GSE26659 (Additional file 1: Table S1) retrieved from the GEO database (https://www.ncbi.nlm.nih.gov/gds), with (∣log2Fold Change (FC)∣ > 1, *p* < 0.05) as the screening threshold to identify the differentially expressed miRNAs. The intersected miRNAs from these 4 datasets were obtained. The expression trend of miRNA in breast cancer was identified based on the expression data of GSE45666 to obtain miRNA closely related to the development of breast cancer. miRNA downstream genes were predicted using the databases including RAID (http://www.rna-society.org/raid2/index.html), TargetScan (Cumulative weighted context +  + score < -0.15; http://www.targetscan.org/vert_71/), mirDIP (Score Class: Very High; http://ophid.utoronto.ca/mirDIP/) and DIANA TOOLS (miTG score > 0.6; http://diana.imis.athena-innovation.gr/DianaTools/). Differential analysis was performed on the breast cancer-related gene expression datasets GSE33447, GSE3744 and GSE50428 (Additional file 1: Table S1) from the GEO database using the R language. Significantly differentially expressed genes were predicted using the GEPIA (http://gepia2.cancer-pku.cn/#index) and TCGA databases (https: //portal.gdc.cancer.gov/). MEM (https://biit.cs.ut.ee/mem/index.cgi) was used to analyze the relationship of co-expression significance of the top 10,000 key downstream genes, and the genes related to key downstream gene expression in GSE50428. LinkedOmics (http://www.linkedomics.org/login.php) was applied to analyze genes related to key downstream gene expression. These genes were intersected and a PPI network was constructed using the String (https://biit.cs.ut.ee/mem/index.cgi). Cytoscape (https://cytoscape.org/) was used to plot and calculate core level of these genes, which were subjected to Kyoto encyclopedia of genes and genomes (KEGG) enrichment analysis. The targeting relationship between miR-182-5p and CMTM7 was predicted using the MICRORNA website (http://www.microrna.org/).

### Clinical sample collection

Cancer and adjacent normal tissue samples were surgically obtained from a total of 40 patients with breast cancer who underwent surgical treatment in our hospital from January 2012 to December 2014. The overall survival was calculated from the surgical resection to the tumor recurrence, and the tumor metastasis was recorded to further collect the metastatic tissue samples. The Edmondson-Steiner grading system was used to determine the histological grade of tumor differentiation (Tumor Pathological Diagnostic Criteria 2016—breast cancer). The tumors were classified according to the World Health Organization (WHO) classification and tumor-lymph node-metastasis (TNM) classification system of the International Union Against Cancer (INM classification of Malignant Tumors, 8th Edition). The cancer and adjacent normal tissues were fixed with formalin and embedded in paraffin for histopathological diagnosis.

### HE staining

For the histopathological assessment, HE staining was performed. The resected primary tumor and lung tissue in nude mouse tumor model were fixed in 10% neutral formalin buffer, embedded and sectioned immediately after surgery. The sections were fixed at room temperature for 30 s, washed with PBS for 2 s, and stained with hematoxylin (60 °C) for 60 s. Following washing with PBS for 10 s, the sections were differentiated with 1% hydrochloric acid alcohol differentiation solution for 3 s, stained with eosin for 3 min, dehydrated in ascending series of alcohol (70%, 80%, 95% and 100%) for 5 min, and cleared in xylene. Finally, the sections were sealed with gum before photograph under an Olympus IX-70 microscope.

### In situ hybridization (ISH) staining

To detect the expression levels of miR-182-5p in tumor and adjacent tissues, ISH assay was performed with the LNA™microRNA ISH miR-182-5p optimization Kit (Exiqon, Woburn, MA). Specifically, on day 1, the slides were placed in xylene for 15 min, and then hydrated to 2 × SSC (AR0058, Boster Biological Technology Co., Ltd., Wuhan, Hubei, China) with ethanol solution, 1 min each time. After incubation in 50 μL of protease K solution (AR0056, Boster) at 37 °C, the slides were washed twice in sterile PBS and dehydrated. Next, 100 μL of Hyb/probe solution was placed on each slide, and Hybridslip cover slip was immediately applied. The slides were successively incubated at 56 °C in a humidity chamber containing 50% formamide/5 × SSC. On day 2, the slides were placed on the vibrating table of RT 2 × SSC for 30 min. After washing in formamide/2 × SSC, 2 × SSC, RNase buffer and 1 × maleate buffer respectively, the slides were incubated with anti-DIG antibody in a humidity chamber. On day 3, the slides were incubated with NBT: BCIP (AR0043, AR0056, Boster) and stained with nuclear fast red. Two pathologists unknown of the patients’ clinicopathological information conducted the scoring according to the staining intensity (strong: 3; moderate: 2; weak: 1; negative: 0) and the abundance of positive cells (≤ 5%: 0; 6–25%: 1; 26–50%: 2; 51–75%: 3; ≥ 76%: 4). The final score obtained by multiplying the intensity score (0–3) with the extent score (0–4) was used to identify the expression of miR-182-5p. The final score 0–4 and 5–12 are defined as low expression and high expression, respectively.

### Immunohistochemical staining

Immunohistochemical staining was used to determine the expression of CMTM7, CD34, CD31, and Ki-67 in the tissues. Cancer tissues were fixed in 4% paraformaldehyde phosphate buffer for 12 h, dewaxed with xylene and hydrated with gradient alcohol (anhydrous ethanol, 95% ethanol and 75% ethanol for 3 min each). The tissues were heated in 0.01 M citrate buffer solution for 15–20 min, and cooled to room temperature, followed by washing with PBS. Thereafter, the tissues were probed with 30 μL primary antibodies against CMTM7 (PA5-103744, 1:200, Thermo Fisher), CD34 primary antibody (ab81289, 1:200, Abcam, Cambridge, UK), CD31 primary antibody (ab134168, 1:100, Abcam) and Ki-67 primary antibody (ab15580, 1:200,Abcam), and then re-probed with 30 μL of secondary antibody goat anti-rabbit IgG (ab6721, 1:2000, Abcam) for 1 h at room temperature. After PBS washing, streptavidin-peroxidase was added dropwise to the tissues and placed at 37 °C for 30 min, followed by DAB development for 5–10 min. Following tap water washing for 10 min, the tissues were counterstaining with hematoxylin for 2 min, differentiated with hydrochloric acid alcohol, dehydrated, cleared and mounted before microscopic observation.

### Microvessel density count (MVD)

The microvessels were stained by CD34 to calculate the MVD according to the previous method (Bao 2018). Under low power fields (40 ×), breast cancer tissue sections were observed under a light microscope (CX33, Olympus Optical Co., Ltd, Tokyo, Japan) to determine the high point of MVD. Then, individual microvessels were counted at 200 × in five regions, and the final result represents the average MVD.

### Cell culture

Human umbilical vein endothelial cells (HUVECs) have the potentiality of stem cells, and are often used for vascular endothelial cell experiments. HUVECs, human normal breast epithelial cell MCF10A and 3 breast cancer cell lines (MCF-7, KPL-4 and MDA-MB-231) (all purchased from the Cell Resource Center of the Institute of Basic Medical Sciences, Chinese Academy of Medical Sciences) were selected for cell experiments in vitro. Following screening, breast cancer cell line MDA-MB-231 and HUVECs were selected for subsequent experiments. HEK-293 T, MCF-7, KPL-4, MDA-MB-231 and MCF-10A cell lines were cultured in DMEM/F12 medium containing 10% fetal bovine serum while HUVECs were cultured in Endothelial Cell Medium (Sciencell, 1001). All cells were cultured in a 37 °C incubator with 5% CO_2_ concentration and 95% saturated humidity, and the cell passage was performed when the cell growth density was about 90%.

### Cell grouping and transfection

MDA-MB-231 and HUVECs were transfected with miR-182-5p mimic, miR-182-5p inhibitor, and their negative controls (NC mimics and NC inhibitor). HUVECs were treated with CMTM7 overexpression vector (oe-CMTM7), DMSO, GDC0068 (AKT inhibitor, 5 nM, Genentech Inc., South San Francisco, USA). The day before transfection, the cells in logarithmic growth phase were seeded into 6-well cell culture plates at a density of 1 × 10^5^ cells/mL. When the cell fusion rate reached 50—70%, 800 μL serum-free medium was added into each well containing cells, and the above-mentioned mimic or inhibitor plasmids (purchased from Bionics Biotech) and lipo2000 mixture (11668027, Thermo Fisher Scientific Inc., Waltham, MA, USA) were separately added to the 6-well plate. After the cells were cultured for 6 h, the new medium was used. After 24-h transfection, Geneticin G418 with the final concentration of 600 mg/L was used for screening and the liquid was changed every 3 days. After 12 days, MDA-MB-231 cells without transfection died, and 48% following transfection still survived. After that, the stably transfected cell line was obtained by expanding the culture under the pressure of 300 mg/L G418.

### Lentiviral transduction

CMTM7-siRNA lentiviral expression vectors (purchased from Genechem company, Shanghai) were cloned into self-inactivated lentivirus vector (GCSIL-NEO). The synthesized oligonucleotides were annealed and inserted into the clone restriction sites of Hind III and EcoR I, and the positive clones were identified by restriction endonuclease cleavage and DNA sequencing. The lentiviral vector and packaging vector (pHelper1.0 and 2.0) with positive inserts were transduced into 293 T cells by Lipofectamine™ (Invitrogen Inc., Carlsbad, CA, USA). From 24 to 72 h after transduction, the supernatant containing lentivirus particles was collected every 12 h and filtered through a 0.45 μm cellulose acetate filter. The final viral titer was 10^9^ tu/mL. One day before transduction, HUVECs were digested with 0.25% trypsin, counted, and seeded into 6-well plates to a density of 2 × 10^5^ cells/well, followed by addition of RPMI-1640 medium containing 10% fetal bovine serum without EVs. The culture was conducted in an incubator with 5% CO_2_ at 37 °C. When the cell fusion rate reached 30–50%, the culture medium in the cells was removed, and 1 mL complete medium with 10 times dilution of virus (dilution ratio was 10^–3^–10^–7^) was added to each well, with addition of the coagel (H8761, Beijing Solarbio Science & Technology Co., Ltd., Beijing, China) to the culture medium. The culture was performed in a 5% CO_2_ incubator at 37 °C. On day 2, the culture medium containing virus was removed, and renewed with 2 mL fresh complete medium, followed by overnight culture. After 5 days, the GFP expression in each group was observed under a fluorescence microscope. The positive rate of GFP was more than 95%. After lentivirus-mediated transduction, stably transduced cells were screened in the medium containing 0.5 μg/ml puromycin.

### EV separation, extraction and identification

MDA-MB-231 cells in logarithmic growth phase were inoculated into MEM medium containing 10% fetal bovine serum without EVs and cultured in cell incubator with 5% CO_2_ at 37 °C. After 2 days, the cell supernatant was collected and filtered with a 0.22 μm filter to remove cell mass. After centrifugation at 300*g* for 10 min at 4 °C, the supernatant was harvested to remove cells. Subsequently, the mixture was centrifuged at 10,000*g* for 30 min to remove cell debris, followed by ultracentrifugation at 110,000*g* for 40 min. The pellets (EVs) were resuspended in 10 ml PBS following ultracentrifugation at 110,000*g* and 4 °C for 90 min. The pellets were stored at − 80 °C after re-suspending with 100 μL PBS. Moreover, EVs were isolated from the serum of 5 patients with breast cancer the same way.

The size distribution of EVs was measured using Nanosizer™ instrument (Malvern instruments, Malvern, UK) dynamic light scattering (DLS), and the morphology of EVs was observed by Hitachi H-7650 transmission electron microscope (TEM, Hitachi, Tokyo, Japan). In brief, EVs were precipitated and immediately fixed in 2.5% glutaraldehyde at 4 °C. After fixation, the specimens were dehydrated using gradient alcohol, embedded in epoxy resin, and cut into ultrathin sections. The sections were stained with uranyl acetate and lead citrate and observed under a TEM (JEM-1010, JEOL Tokyo, Japan). The expression of the EVs surface biosignature proteins CD63, Hsp70, TSG101 and Calnexin was detected by Western blot analysis.

### Co-culture of EVs and HUVECs

HUVECs in logarithmic growth phase were seeded in a 6-well plate at a density of 5 × 10^5^ cells/well with RPMI-1640 medium containing 10% FBS. EVs isolated from MDA-MB-231 cells were co-cultured with HUVECs at the concentration of 100 μg/mL for 3 days. HUVECs were cultured in the serum-free medium for 12 h before stimulation experiment.

MDA-MB-231 cells in logarithmic growth phase were placed in a Transwell chamber with polycarbonate (PC), polyester (PET) or collagen coated polytetrafluoroethylene (PTFE) membrane at a density of 5 × 10^5^ cells/well. (The membrane at the bottom of the Transwell chamber separated the upper and lower cells. The diameter of the membrane was less than 3.0 μm, generally 0.4 μm. The cells cannot pass through the membrane freely, but cytokines can, and the two can exchange information through this membrane.) Then, the Transwell chamber was placed in a culture plate containing HUVECs (5 × 10^5^ /well) for 3-day co-culture. Then HUVECs were collected for experiment. Meanwhile, GW4869 (EV release inhibitor) treatment group was designed.

### EVs labeling

To explore whether EVs can transfer miR-182-5p into HUVECs, FAM (green)-labeled miR-182-5p was transfected into MDA-MB-231 cells, and the isolated EVs containing FAM-miR-182-5p were labeled with Dil (red). Then the EVs were co-cultured with Hoechst (blue) labeled HUVECs. The intracellular fluorescence signals were observed under a fluorescence microscope.

### Tracking analysis of EVs

The collected EVs were diluted with PBS to the particle concentration of 10^6^/mL–10^9^/mL, and the sample was taken with a 1 mL syringe and injected into the Nanosight analyzer (Nanosight NS300, Malvern, UK) for detection and analysis.

### EV internalization by HUVECs

To observe the uptake of EVs by HUVECs, EVs were labeled with green fluorescent dye (PKH67; Sigma) and co-cultured with HUVECs at 37 °C for 2 h. The cells were washed with PBS, fixed with 4% paraformaldehyde for 15 min and stained with Hoechst. The intracellular green fluorescence was observed by a fluorescence microscope (CX23, Olympus). The receptor cells stimulated by EVs were collected, and the expression of miRNA was detected by RT-qPCR.

### RT-qPCR

TRIzol reagent (15596-018, Solarbio) was used to extract total RNA from tissues or cells and the RNA concentration was determined. The primers used in this study were synthesized by Takara company (Dalian, China) (Additional file 1: Table S2). The extracted total RNA was reverse transcribed into cDNA according to the instructions of one-step miRNA reverse transcription kit (D1801, Harbin HaiGene Biotech Co., Ltd., Harbin, China) and cDNA reverse transcription kit (K1622, Beijing Yaanda Biotechnology Co., Ltd., Beijing, China). RT-qPCR was performed on a fluorescence quantitative PCR instrument (ViiA 7, Sun Yat Sen University, Daan gene Co., Ltd., UK). U6 and GAPDH were used as internal references, and the relative transcription level of target genes was calculated by relative quantitative method (2^−ΔΔCT^ method).

### Western blot analysis

The total protein was extracted from tissues or cells with radioimmunoprecipitation assay lysis buffer (R0010, Solarbio), with the concentration determined by BCA Kit (20201ES76, YEASEN Biotech Co., Ltd., Shanghai). After separation by polyacrylamide gel electrophoresis, the protein was transferred to the PVDF membrane by wet transfer method. The membrane was sealed with 5% BSA at room temperature for 1 h, probed with the primary antibodies to CMTM7 (#PA5-103744, 1:1000, Thermo Fisher), EGFR (ab52894, 1:1000, Abcam), p-EGFR (ab40815, 1:1000, Abcam), AKT (ab8805, 1:500, Abcam), p-AKT (ab8933, 1:500, Abcam), VEGF (ab32152, 1:1000), CD63 (ab134045, 1:1000, Abcam), Hsp70 (ab181606, 1:1000, Abcam), TSG101 (ab125011, 1:2000, Abcam), and Calnexin (ab133615, 1:1000, Abcam) at 4 °C overnight. The next day, the membrane was re-probed with HRP labeled goat anti-rabbit IgG (ab205718, 1:10,000, Abcam) for 1 h at room temperature, developed by VILBER FUSION FX5 (VILBER LOURMAT, France). Image J 1.48u software (National Institutes of Health) was used for protein quantitative analysis, and the gray value of each protein was compared with the gray value of internal reference GAPDH.

### Dual-luciferase reporter assay

Dual-luciferase reporter assay was applied to verify the interaction between miR-182-5p and CMTM7. The 3'UTR dual-luciferase reporter gene vector of CMTM7 and the mutant plasmid of miR-182-5p binding site mutation were constructed respectively: PmirGLO-CMTM7-WT and PmirGLO-CMTM7-MUT. The reporter plasmid was co-transfected into MDA-MB-231 cells with miR-182-5p mimic and mimic NC, respectively. The cells were lysed after 24 h of transfection, and the supernatant was collected by centrifugation at 13,000*g* for 1 min. The luciferase activity was detected by Dual-Luciferase® Reporter Assay System (E1910, Promega Corporation, Madison, WI, USA). The activity of renilla luciferase was taken as the internal reference, and the relative luciferase activity was expressed as the ratio of firefly luciferase activity to renilla luciferase activity.

### CCK-8 assay

CCK-8 kit (WH1199, Shanghai Weiao Biotechnology Co., Ltd., Shanghai, China) was used to detect cell proliferation. The cells in logarithmic growth phase were adjusted to a concentration of 5 × 10^4^ cells/mL using RPMI-1640 medium (Gibco, USA) containing 10% FBS, and seeded in 96-well culture plates. Then 100 μL cell medium was added to each well, and the culture was performed in an incubator for 20, 40, 60 and 80 h. The supernatant was quickly discarded, and 10 μL CCK-8 solution was added into each well. After culture at 37 °C for 2 h, the absorbance value (A) was determined by Multiskan FC microplate reader (51119080, Thermo Fisher Scientific) at a wavelength of 450 nm, and the proliferation rate (%) = [(A _Control group_—A _Experimental group_) / A _Control group_] × 100%. Three parallel wells were set in each group, and the average value was taken.

### Scratch test

Migration ability of MDA-MB-231 cells and HUVECs after transfection was accessed using the scratch test. The required ruler and marker pen were sterilized by ultraviolet irradiation for 30 min before operation. Approximately 5 × 10^5^ cells were seeded into a 6-well plate and cultured overnight at 37 °C. The next day, 10 μL pipette tip against the ruler was used to draw two parallel lines perpendicular to the mark line, and the pipette tip should be vertical and not inclined. Following three washes with PBS, the scratched cells were removed. Serum-free medium was added to the cells and cultured in a 37 °C incubator after which the cell growth state was observed under a microscope at 0 h and 48 h, and photographed.

### Transwell migration assay

Transwell assay was performed to detect the migration ability of MDA-MB-231 cells and HUVECs after transfection. The cells were digested, counted and prepared into cell suspension with complete medium. In each well, 1 × 10^5^ cells/μL and 800 μL conditioned medium containing 20% FBS were added into the upper chamber and the lower chamber, respectively, followed by culture in a 37 °C incubator for 16 h. Transwell plate was taken out, rinsed twice with PBS, immersed in formaldehyde for 10 min, stained with 0.1% crystal violet, and left to stand at room temperature for 30 min. Following two washes with PBS, the cells on the upper surface were wiped off with cotton balls. Finally, the cells were observed, photographed and counted under an inverted microscope.

### Vessel-like tube formation assay

The capillary-like structures formed by HUVECs were determined by tube formation assay. One day before the experiment, Matrigel was placed in an ice box and thawed overnight in a 4 °C refrigerator. The 24-well plate, sterilized pipette tips for Matrigel, and 1.5 mL EP tube were placed in − 20 °C refrigerator. The tube was removed and Matrigel was packed according to the dosage. Next, the refrigerated 24-well plate and pipette tips were taken out, and 200 μL Matrigel was added into each well of the plate. The 24-well plate covered with Matrigel was placed in the incubator at 37 °C for 30 min to form gel, while the single cell suspension of treated HUVECs was prepared and counted. After Matrigel was solidified, 1 × 10^4^ cells were seeded into 24-well plates, shaken well (without bubbles) and cultured in a 37 °C incubator for 4–6 h. After culture, the vessel-like tube formation was observed under a microscope, with the images collected regularly. The tube length and node number were measured and recorded.

### Xenograft tumor assay in nude mice

Sixty five-to-seven-week-old BALB/c nude mice (irrespective of gender; provided by Shanghai Experimental Animal Center, Chinese Academy of Sciences, Shanghai, China) were fed with fine pellets, and given ad libitum access to drinking water, and housed under conditions of natural light with 12-h light/dark cycle. MDA-MB-231 cells were subcutaneously inoculated into mice at a density of 1 × 10^6^, and the remaining 10 were used as the control. The maximum diameter (L) and minimum diameter (W) of the tumor were measured with a vernier caliper every 1 week, and the tumor volume was calculated using the formula: V = W^2^ × L × 0.5. When the tumor volume reached 50 mm^3^, 50 nude mice were randomly divided into 5 groups, with 10 mice in each group: EVs + inhibitor NC (mice injected with 25 μg of EVs isolated from MDA-MB-231 cells transfected with NC via tail vein), EVs + miR-182-5p inhibitor (mice injected with 25 μg of EVs isolated from MDA-MB-231 cells transfected with miR-182-5p inhibitor via tail vein), EVs + miR-182-5p inhibitor + si-NC (mice injected with EVs isolated from MDA-MB-231 cells transfected with NC and 25 μg of EVs isolated from MDA-MB-231 cells transfected with miR-182-5p inhibitor via tail vein), EVs + miR-182-5p inhibitor + si-CMTM7 (mice injected with EVs isolated from MDA-MB-231 cells transfected with si-CMTM7 and 25 μg of EVs isolated from MDA-MB-231 cells transfected with miR-182-5p inhibitor via tail vein). The control mice were injected with normal saline via tail vein. Among them, EVs were injected every 3 days for 4 weeks. Tumor volume was calculated every 3 days after one week of injection. When the weight loss of the nude mice exceeded 10% or the tumor volume exceeded 1200 mm^3^, the nude mice were euthanized, and the tumors were removed and weighed. At the same time, the lung tissues of nude mice were collected from each group and the metastasis of tumors was observed and detected, with the number of tumor cells counted.

### Statistical analysis

All data were processed by SPSS 21.0 statistical software (IBM Corp. Armonk, NY, USA). The measurement data were expressed as mean ± standard deviation. Data between two groups were compared using independent sample *t*-test. Comparison of data among multiple groups was conducted by one-way analysis of variance (ANOVA), followed by Tukey's post hoc tests with corrections for multiple comparisons. The repeated measures ANOVA with Bonferroni post hoc test was applied for the comparison of data at different time points. Pearson correlation coefficient is used to evaluate the relationship between miR-182-5p and MVD. The statistical power was calculated with the G*Power software (Ver. 3.1.9.6). The Kaplan–Meier method was used to calculate the survival rates and the differences between the survival curves were examined using the log-rank test. *p* < 0.05 indicated the difference was statistically significant.

## Results

### miR-182-5p is highly expressed in breast cancer tissues and cells, which is associated with poor prognosis of breast cancer patients

**Fig. 1:**
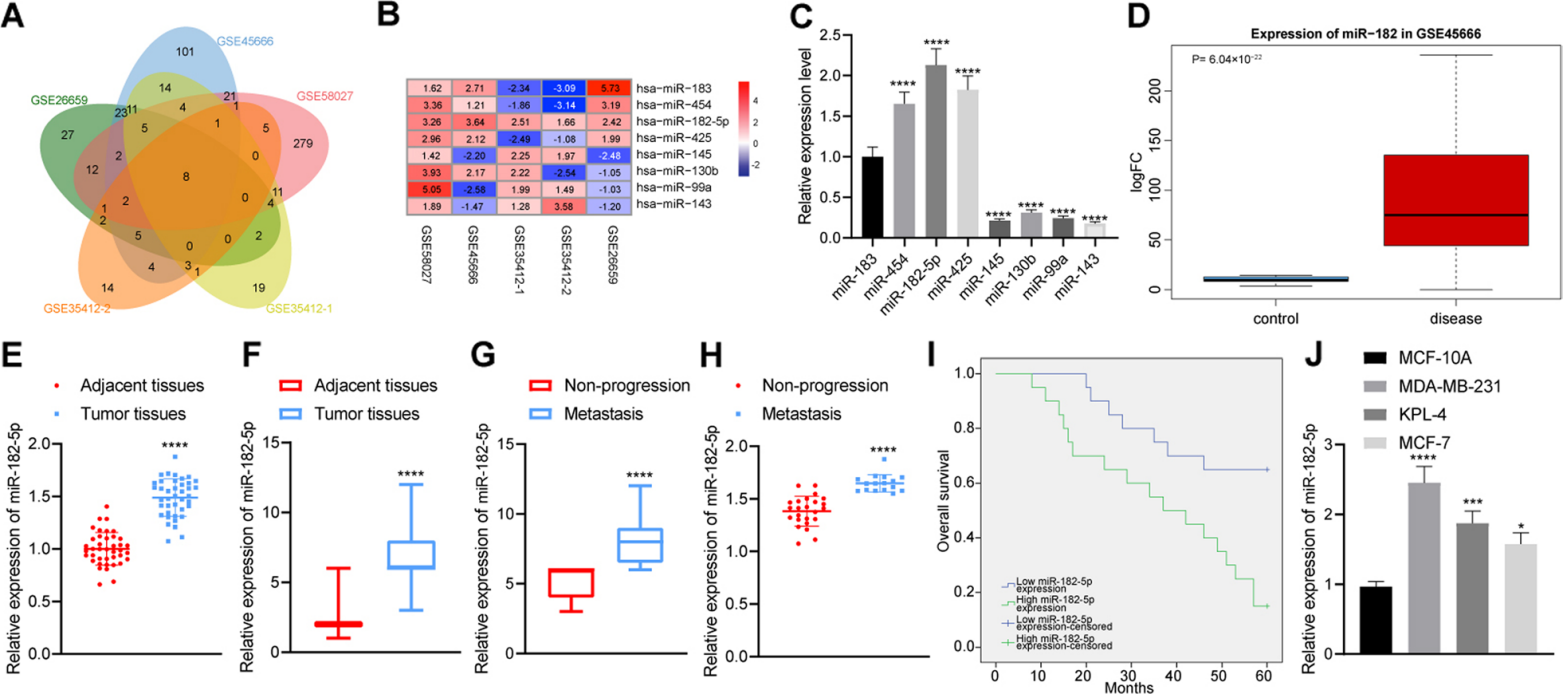
Upregulated expression of miR-182-5p in breast cancer tissues and cells. **A** Venn diagram of the identified differential miRNAs in breast cancer-related datasets GSE26659, GSE35412-1, GSE35412-2, GSE45666 and GSE58027; **B** A heat map of 8 intersected miRNA expression in breast cancer-related datasets GSE26659, GSE35412-1, GSE35412-2, GSE45666 and GSE58027 (the right shows the color scale of log2FC value); **C** Expression of 8 intersected miRNAs (miR-183, miR-454, miR-425, miR-145, miR-130b, miR-99a, miR-143, miR-182-5p) in MDA-MB-231 cells detected by RT-qPCR, the statistical power was 1; **D** A box plot of miR-182-5p expression in breast cancer tissue and adjacent normal tissue in the dataset GSE45666 (the left blue box represents the expression of adjacent normal tissue samples, and the right red box represents the expression of breast cancer tissue samples); **E** Expression of miR-182-5p in cancer tissues and adjacent normal tissues of 40 breast cancer patients detected by RT-qPCR, the statistical power was 1; **F** Expression of miR-182-5p in breast cancer tissues and adjacent normal tissues detected by ISH, the statistical power was 1; **G** Expression of miR-182-5p in metastasis tissues of 16 breast cancer patients with distant metastasis and cancer tissue of 24 breast cancer patients without distant metastasis detected by ISH, the statistical power was 1; **H** Expression of miR-182-5p in metastasis tissues of 16 breast cancer patients with distant metastasis and cancer tissue of 24 breast cancer patients without distant metastasis detected by RT-qPCR, the statistical power was 1; **I** Correlation between the expression of miR-182-5p and the prognosis of 40 breast cancer patients analyzed by Kaplan–Meier curve analysis; **J** Expression of miR-182-5p in 4 cell lines (MCF-10A, MCF-7, KPL-4 and MDA-MB-231) detected by RT-qPCR, the statistical power was 1. *p < 0.05, ***p < 0.001, ****p < 0.0001 compared with adjacent normal tissues, breast cancer patients without distant metastasis or MCF-10A cell line. The experiment was conducted three times independently

Analysis on the GSE26659, GSE35412-1, GSE35412-2, GSE45666 and GSE58027 (Fig. 1A, B) revealed 104, 83, 47, 205 and 356 differentially expressed miRNAs, respectively. Eight miRNAs (miR-183, miR-454, miR-425, miR-145, miR-130b, miR-99a, miR-143, miR-182-5p) were found at the intersection. The expression of the eight differentially expressed miRNAs in MDA-MB-231 cells was detected by RT-qPCR. The results showed that the expression of miR-182-5p was the highest (Fig. 1C). GSE45666 found that the expression of miR-182-5p was significantly higher in breast cancer tissue samples than that in adjacent normal tissue samples (Fig. 1D).

Similarly, RT-qPCR data revealed that the expression of miR-182-5p was significantly higher in clinical breast cancer tissues than that in adjacent normal tissues (Fig. 1E). The results of ISH showed that the expression of miR-182-5p in breast cancer tissue samples was elevated compared to adjacent normal tissues (Fig. 1F, Additional file 2: Fig. S1), and the expression of miR-182-5p in breast cancer tissues with distant metastasis was significantly higher than that in breast cancer tissues without distant metastasis (Fig. 1G, H). Kaplan–Meier analysis using the log-rank test showed that the overall survival of breast cancer patients with high miR-182-5p expression was worse than those with low expression of miR-182-5p (Fig. 1I). The RT-qPCR results showed that, compared with human normal mammary epithelial cell MCF-10A, miR-182-5p was abundant in three breast cancer cell lines (MCF-7, KPL-4 and MDA-MB-231), of which MDA-MB-231 cells proved to have the most abundant miR-182-5p (Fig. 1J). The above results demonstrated that miR-182-5p was highly expressed in breast cancer tissues and cells, and high expression of miR-182-5p was related to poor prognosis of breast cancer patients.

### miR-182-5p promotes tumor angiogenesis by enhancing angiogenesis of HUVECs

**Fig. 2:**
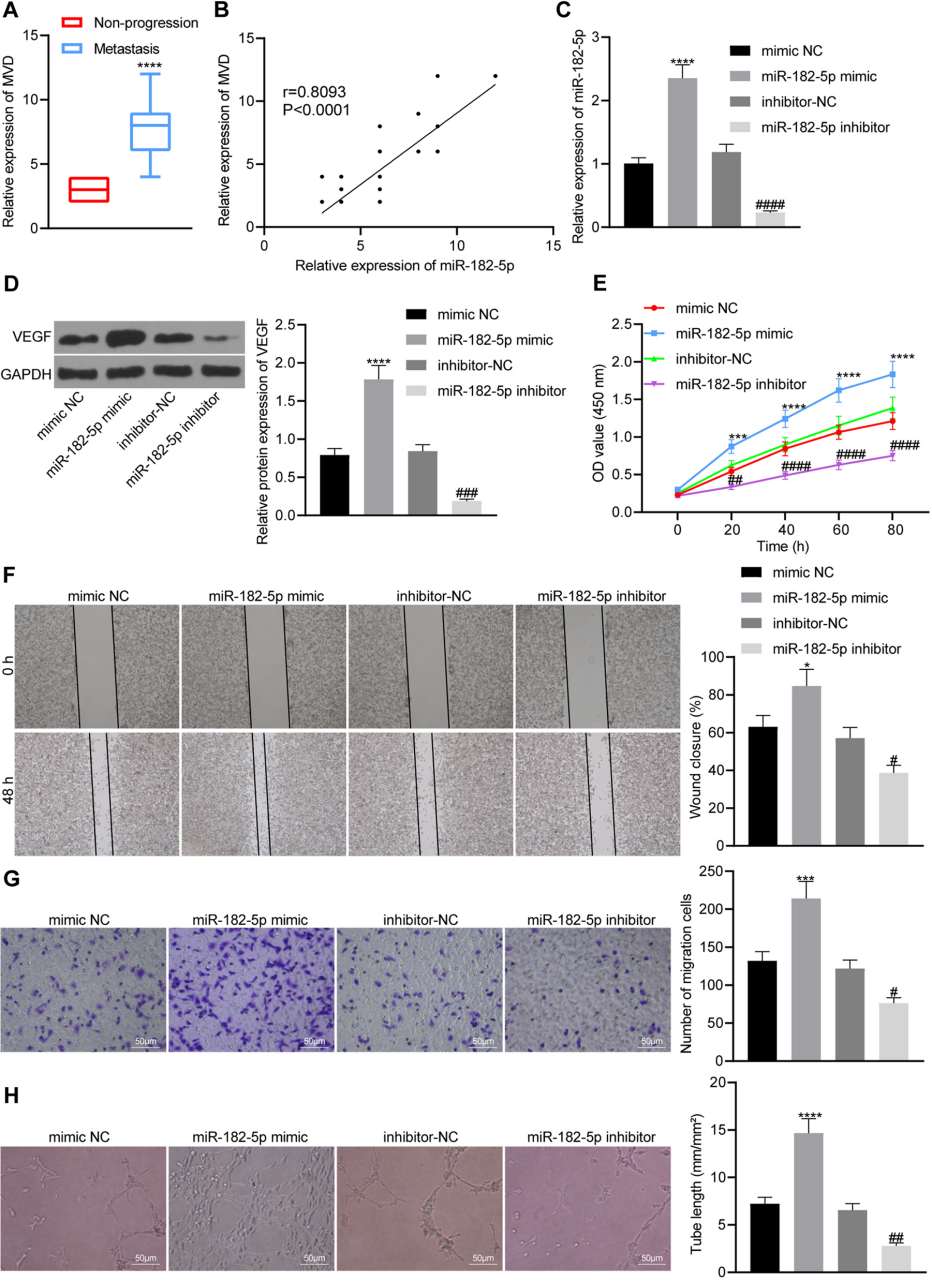
miR-182-5p stimulates proliferation, migration and angiogenesis of HUVECs. **A** miR-182-5p expression and MVD in metastasis tissues from 16 breast cancer patients with distant metastasis detected by ISH and immunohistochemical staining, the statistical power was 1; **B** Pearson correlation analysis of the relationship between miR-182-5p and MVD, the statistical power was 0.572; **C** miR-182-5p expression in HUVECs detected by RT-qPCR, the statistical power was 1; **D** VEGF protein expression in HUVECs detected by Western blot analysis, the statistical power was 1; **E** Proliferation of HUVECs detected by CCK-8 method, the statistical power was 1; **F** HUVECs migration ability detected by Scratch test, the statistical power was 1; **G** HUVECs migration ability detected by Transwell assay, the statistical power was 1; **H** Vessel-like tube formation in HUVECs, the statistical power was 1. *p < 0.05, ***p < 0.001, ****p < 0.0001 compared with cells treated with mimic NC or breast cancer patients without distant metastasis; #*p* < 0.05, ##*p* < 0.01, ###*p* < 0.001, ####*p* < 0.0001 compared with cells treated with inhibitor NC. The experiment was conducted three times independently

As shown in Fig. 2A, the MVD was significantly higher in breast cancer tissues with distant metastasis than in the tissues without distant metastasis. Meanwhile, Pearson correlation analysis showed positive correlation of the expression of miR-182-5p with MVD in metastasis tissues of breast cancer patients (Fig. 2B). Therefore, we suspected that miR-182-5p may promote breast cancer tumor angiogenesis. RT-qPCR results (Fig. 2C) further showed that miR-182-5p mimic and miR-182-5p inhibitor were successfully transfected into HUVECs. Western blot analysis (Fig. 2D) showed that miR-182-5p mimic increased the expression of VEGF protein in HUVECs, while miR-182-5p inhibitor reduced the expression. The results of CCK-8, scratch test and Transwell assay showed that miR-182-5p mimic promoted HUVECs proliferation, and migration, which was negated by miR-182-5p inhibitor (Fig. 2E–G). Figure 2H revealed that miR-182-5p mimic enhanced the in vitro vessel-like tube formation, while miR-182-5p inhibitor resulted in a contrary trend. The above results demonstrated that miR-182-5p can promote breast cancer tumor angiogenesis by enhancing the proliferation, migration and angiogenesis of HUVECs.

### Breast cancer cell-derived EVs transfer miR-182-5p to HUVECs

**Fig. 3:**
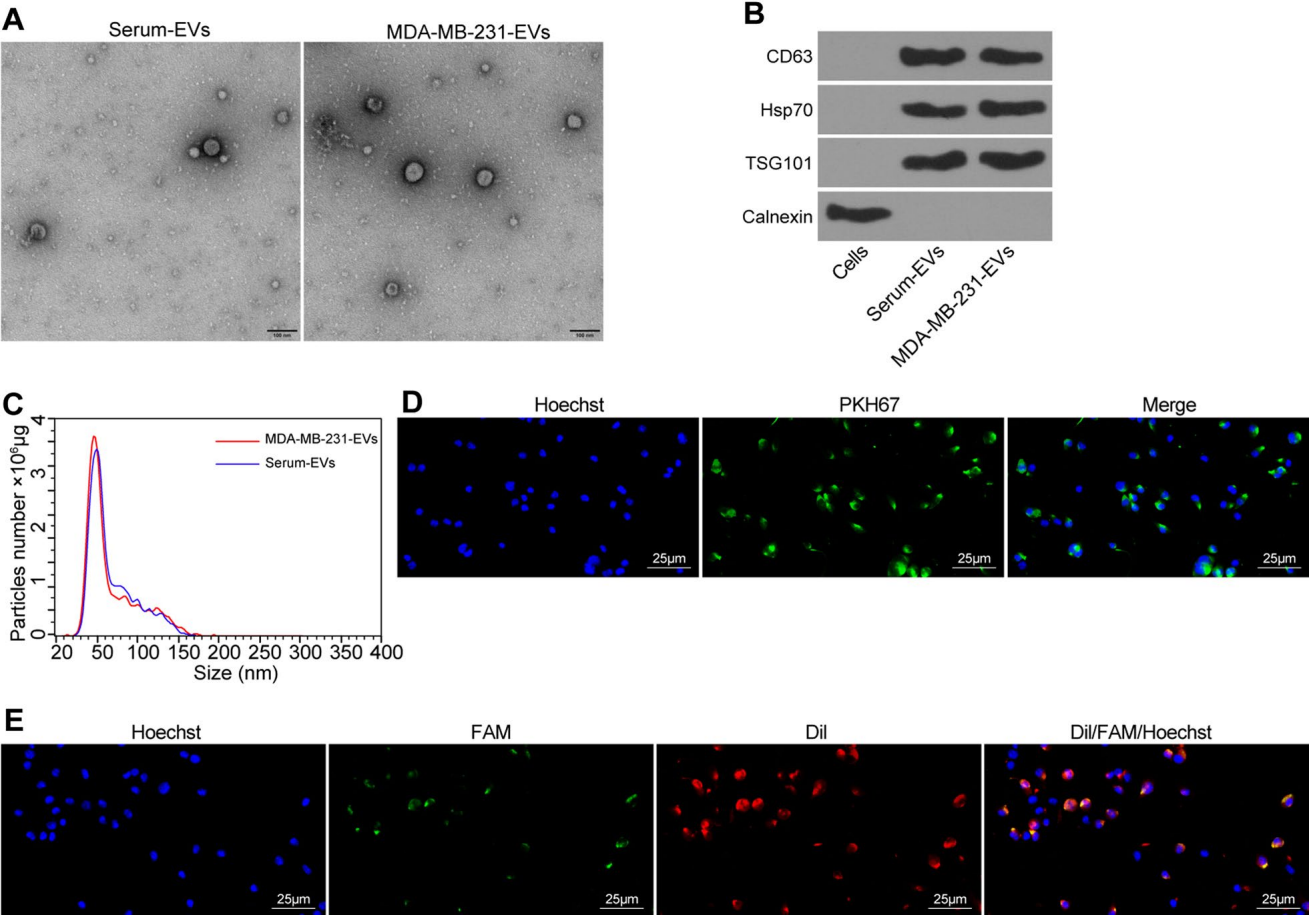
EVs derived from breast cancer cells transfer miR-182-5p to HUVECs. **A** Morphological characteristics of EVs derived from the serum of breast cancer patients and MDA-MB-231 cells under a TEM; **B** Expression of EV marker proteins CD63, Hsp70, TSG101 and Calnexin detected by Western blot analysis; **C** Diameter of EVs detected by Nanosight Nanoparticle Tracking Analyzer; **D** Uptake of EVs in HUVECs under a confocal microscope, blue: Hoechst staining, green: PKH67-labeled EVs; **E** miR-182-5p transfer by EVs to HUVECs detected by immunofluorescence (green fluorescent labeled miR-182-5p, red fluorescent labeled EVs, blue fluorescent represented the nucleus). The experiment was conducted three times independently

Under the TEM, a group of round or oval small membranous vesicles with obvious heterogeneity can be observed in the EVs isolated from the serum of patients as well as MDA-MB-231 cells. The membranous structure could be found on the periphery of the vesicle, and the component with low electron density was shown in the middle (Fig. 3A).

Western blot analysis results showed that the EV marker proteins CD63, Hsp70 and TSG101 were positive, but Calnexin was not expressed (Fig. 3B). Meanwhile, EVs exhibited irregular Brownian motion with a diameter of 30–100 nm (Fig. 3C). Since no obvious difference was observed between the EVs from patient serum and MDA-MB-231 cells, we then explored the effect of breast cancer cell-derived EVs on endothelial cells by co-culturing HUVECs with PKH-67-labeled EVs for 2 h. As a result, HUVECs exhibited high efficiency of EV uptake (Fig. 3D). The above results indicated the successful isolation of EVs, and that EVs derived from breast cancer cells could be internalized by HUVECs.

In order to explore whether EVs can transfer miR-182-5p into HUVECs, we first transfected FAM (green) labeled miR-182-5p into MDA-MB-231, and then labeled EVs containing FAM -miR-182-5p with Dil (red), which were then co-cultured with Hoechst (blue)-labeled HUVECs. The fluorescence microscope showed that red and green signals could be detected simultaneously in the cytoplasm of HUVECs co-cultured with EVs (Fig. 3E). The above results demonstrated that EVs derived from breast cancer cells delivered miR-182-5p to HUVECs.

### miR-182-5p delivered by EVs (EVs-miR-182-5p) enhances the migration, proliferation and angiogenesis of HUVECs

**Fig. 4:**
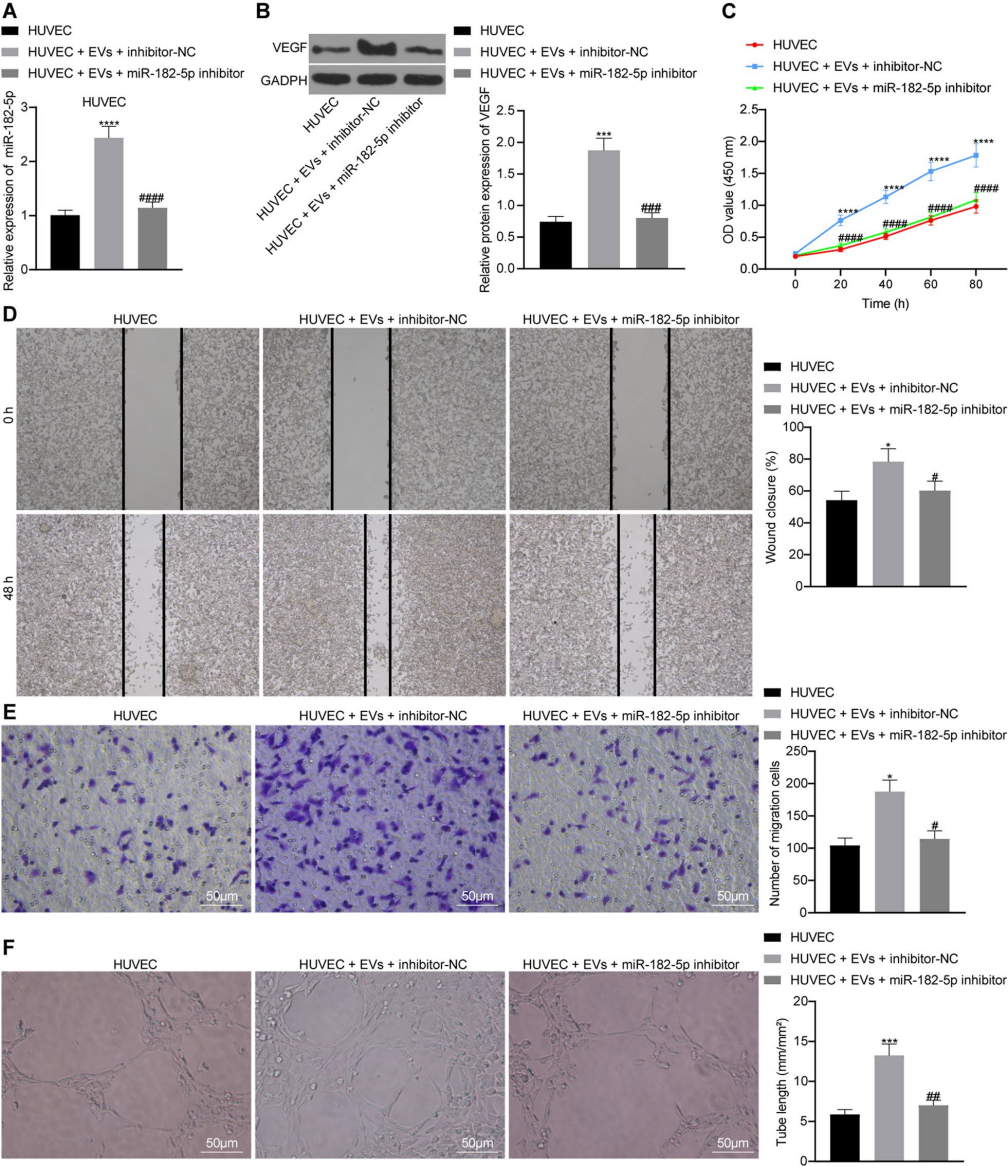
EVs-miR-182-5p exerts promoting effects on HUVECs proliferation, migration and angiogenesis. HUVECs were treated with EVs + miR-182-5p inhibitor. **A** miR-182-5p expression of HUVECs detected by RT-qPCR, the statistical power was 1; **B** VEGF protein expression of HUVECs detected by Western blot analysis, the statistical power was 1; **C** The proliferation of HUVECs detected by CCK-8 assay, the statistical power was 1; **D** Migration ability of HUVECs detected by scratch test, the statistical power was 1; **E** Migration ability of HUVECs detected by Transwell assay, the statistical power was 1; **F** Vessel-like tube formation in vitro of HUVEC, the statistical power was 1. *p < 0.05, ***p < 0.001, ****p < 0.0001 compared with HUVEC; #*p* < 0.05, ##*p* < 0.01, ###*p* < 0.001 compared with HUVEC treated with EVs + inhibitor NC. The experiment was conducted three times independently

We co-cultured HUVECs with EVs after different treatments to explore whether EVs-miR-182-5p affects the angiogenesis of HUVECs. The expression of miR-182-5p was increased by EVs treatment and reduced following miR-182-5p inhibitor transfection (Fig. 4A). Moreover, Western blot analysis results showed that the expression of VEGF protein was increased with EVs treatment and reduced in the absence of miR-182-5p (Fig. 4B). The inhibition of miR-182-5p attenuated the proliferation and migration of HUVECs (Fig. 4C–E). In addition, as depicted in Fig. 4F, vessel-like tube formation in vitro was weakened in following transfection with miR-182-5p inhibitor. Taken together, EVs-miR-182-5p enhanced the migration, proliferation along with angiogenesis of HUVECs, and miR-182-5p inhibition reversed the effect.

### miR-182-5p targets CMTM7 in breast cancer cells

**Fig. 5:**
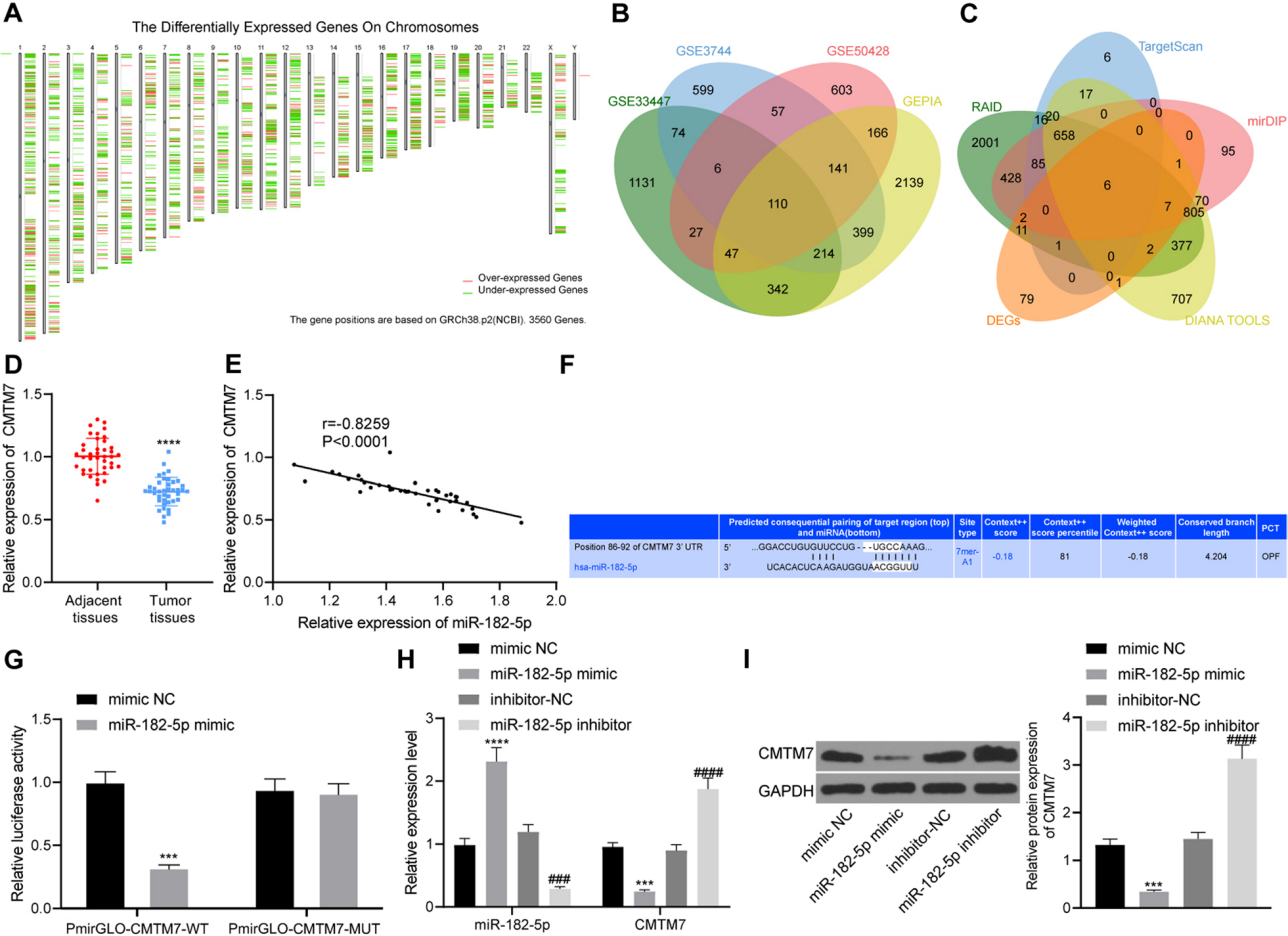
CMTM7 is a target of miR-182-5p. **A** A heat map of differential gene expression in breast cancer samples from the TCGA database by GEPIA (from left to right, chromosomes 1–22 and sex chromosomes are present in sequence); **B** Venn diagram of differential genes screened from datasets GSE33447, GSE3744 and GSE50428 in GEO database and GEPIA; **C** Venn diagram of miR-182-5p downstream genes and significantly differential genes predicted by RAID, TargetScan, mirDIP and DIANA TOOLS databases; **D** CMTM7 expression in cancer tissues and adjacent normal tissues of 40 breast cancer patients detected by RT-qPCR, he statistical power was 1; **E** Pearson correlation analysis of the expression of miR-182-5p and CMTM7 in breast cancer tissues, the statistical power was 0.999; **F** Binding site between miR-182-5p and CMTM7 predicted by the TargetScan website; **G** Binding of miR-182-5p to CMTM7 confirmed by dual luciferase experiment, the statistical power was 1; **H** Expression of miR-182-5p and CMTM7 of HUVECs detected by RT-qPCR, the statistical power was 1; **I** Expression of CMTM7 protein of HUVECs detected by Western blot analysis, the statistical power was 1. ***p < 0.001, ****p < 0.0001 compared with adjacent normal tissues, or HUVECs treated with mimic NC. ###*p* < 0.001, ####*p* < 0.0001 compared with HUVECs treated with inhibitor NC. The experiment was conducted three times independently

In order to further explore the downstream target genes of miR-182-5p, we analyzed the datasets GSE33447, GSE3744 and GSE50428 and identified 1951, 1600 and 1157 differentially expressed genes, respectively. Moreover, 3558 differentially expressed genes were screened in breast cancer samples in TCGA database by GEPIA analysis (Fig. 5A). Intersection analysis suggested 110 key differential genes (Fig. 5B). In addition, RAID, TargetScan, mirDIP and DIANA TOOLS databases predicted 4419, 809, 2157 and 2671 downstream genes of miR-182-5p, respectively, with 6 key downstream genes were confirmed at the intersection (Fig. 5C), including CMTM7. Published data showed that CMTM7 was poorly expressed in several tumors, including colorectal cancer, pancreatic cancer and esophageal cancer, involved in the occurrence and metastasis of a variety of tumors (Jin et al. 2018; Huang 2019; Li 2014). The RT-qPCR results showed that the expression of CMTM7 was down-regulated in breast cancer tissues compared with adjacent normal tissues (Fig. 5D). Pearson correlation analysis confirmed that miR-182-5p and CMTM7 were negatively correlated in breast cancer tissues (Fig. 5E).

TargetScan website predicted binding sites at 86–92 between miR-182-5p and CMTM7 (Fig. 5F). Meanwhile, the results of dual luciferase experiment showed that fluorescence intensity of PmirGLO-CMTM7-WT was inhibited in response to miR-182-5p mimic while no difference was observed in fluorescence intensity of PmirGLO-CMTM7-MUT (Fig. 5G). The results of RT-qPCR and Western blot analysis showed that the expression of miR-182-5p was increased while that of CMTM7 was reduced in response to miR-182-5p mimic. However, in the absence of miR-182-5p, these results were reversed (Fig. 5H, I). The above results demonstrated that miR-182-5p targeted CMTM7 and caused the inhibition of CMTM7 expression in breast cancer cells.

### EVs-miR-182-5p targets CMTM7 and activates the EGFR/AKT signaling pathway to promote breast cancer angiogenesis

We co-cultured HUVECs with EVs after different treatments to explore whether CMTM7 affects the angiogenesis of HUVECs. The expression of CMTM7 was increased following transfection with oe-CMTM7, while treatment with EVs suppressed the CMTM7 expression (Additional file 3: Fig. S2A–B). Overexpression of CMTM7 attenuated the proliferation, migration, and vessel-like tube formation of HUVECs (Additional file 3: Fig. S2C–F). Moreover, CMTM7 overexpression could abolish the effects of EVs treatment on HUVECs.

**Fig. 6:**
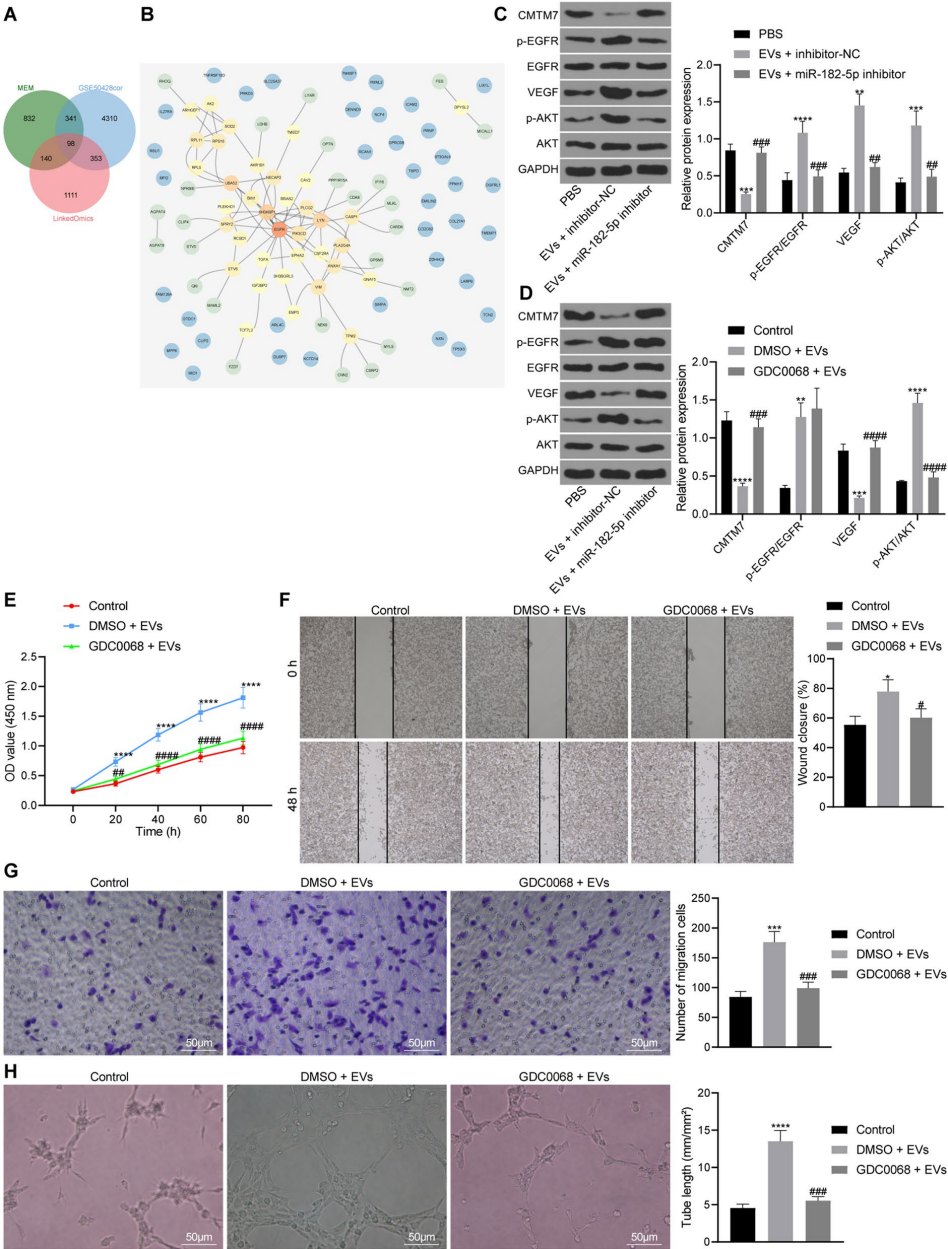
EVs-miR-182-5p induces proliferation, migration and angiogenesis of HUVECs through inhibition of CMTM7 and activation of the EGFR/AKT signaling pathway. **A** Venn diagram of genes related to CMTM7 expression in breast cancer tissue samples from dataset GSE50428, MEM database and LinkedOmics database; **B** PPI network of 98 intersection genes related to CMTM7 expression constructed using String (in the PPI network, the higher core degree is, the more red the circle color is; otherwise, the lower the core degree, the bluer the color is); **C** Expression of CMTM7, VEGF, p-EGFR, EGFR, p-AKT and AKT in HUVECs treated with EVs or combined with miR-182-5p inhibitor by Western blot analysis, the statistical power was 1; **D** Expression of CMTM7, VEGF, p-EGFR, EGFR, p-AKT and AKT in HUVECs treated with EVs or combined with GDC0068 detected by Western blot analysis, the statistical power was 1; **E** Proliferation of HUVECs treated with EVs or combined with GDC0068 detected by CCK-8 method, the statistical power was 1; **F** Migration of HUVECs treated with EVs or combined with GDC0068 detected by scratch test, the statistical power was 1; **G** Migration ability of HUVECs treated with EVs or combined with GDC0068 detected by Transwell assay, the statistical power was 1; **H** Vessel-like tube formation in vitro of HUVECs treated with EVs or combined with GDC0068, the statistical power was 1. *p < 0.05, **p < 0.01, ***p < 0.001, ****p < 0.0001 compared with control HUVECs, or HUVECs treated with PBS or EVs + inhibitor NC. #*p* < 0.05, ##*p* < 0.01, ###*p* < 0.001 compared with HUVECs treated with DMSO-EVs. The experiment was conducted three times independently

Then, in order to explore the specific molecular mechanism of CMTM7 participating in breast cancer angiogenesis, we screened out 5102 genes related to CMTM7 expression in the GSE50428 dataset, and predicted 1411 genes co-expressed with CMTM7 through the MEM database. The LinkedOmics database predicted 1702 genes related to the expression of CMTM7 in breast cancer tissues in the TCGA database, and 98 intersected genes were obtained following Venn diagram analysis (Fig. 6A). PPI network of the 98 intersected genes was constructed using STRING, showing that EGFR had the highest core degree (Fig. 6B). CMTM7 can promote EGFR internalization and further inhibit activation of the AKT pathway (Liu 2015). Moreover, the EGFR/AKT signaling pathway is closely related to tumor angiogenesis (Jin 2011). Therefore, we speculated whether EVs-miR-182-5p can regulate activation of the EGFR/AKT signaling pathway through inhibition of CMTM7, thus promoting breast cancer angiogenesis.

For validation, we first co-cultured EVs with HUVEC, and then detected the expression of VEGF and EGFR/AKT signaling pathway-related proteins in HUVECs by Western blot analysis. The results showed that the expression of CMTM7 was reduced, but EGFR and AKT phosphorylation levels were increased in the HUVECs treated with EVs + inhibitor NC, the effect of which abolished in the presence of EVs + miR-182-5p inhibitor (Fig. 6C).

As shown in Fig. 6D, the expression of CMTM7 was reduced in HUVECs treated with DMSO + EVs while EGFR and AKT phosphorylation levels were significantly increased. Besides, no significant change was shown in CMTM7 and EGFR phosphorylation level in the presence of GDC0068 + EVs but AKT phosphorylation level was reduced. Additionally, the proliferation and migration of HUVECs in the presence of EVs were increased but combined treatment with GDC0068 and EVs led to an opposite result (Fig. 6E–G). Figure 6H showed that the vessel-like tube formation of HUVECs increased significantly in response to treatment with EVs while further GDC0068 treatment negated this trend. Overall, EVs-miR-182-5p activated the EGFR/AKT signaling pathway and enhanced the proliferation, migration and angiogenesis of HUVECs through inhibition of CMTM7.

### EVs-miR-182-5p promotes tumorigenesis and metastasis in vivo by regulating the CMTM7/EGFR/AKT axis

**Fig. 7:**
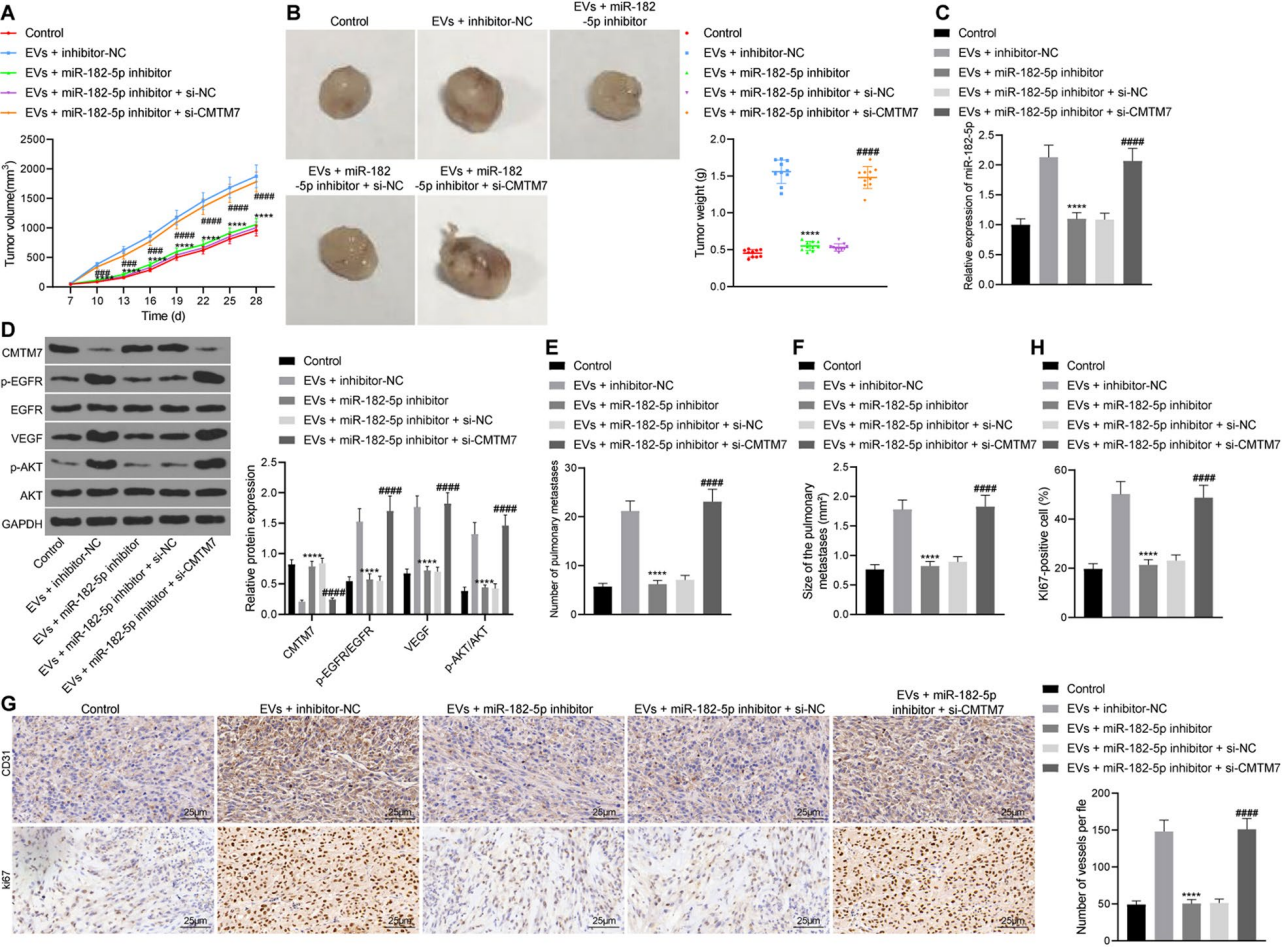
EVs-miR-182-5p exerts promoting effect on tumorigenesis and metastasis of breast cancer cells in vivo by the CMTM7/EGFR/AKT axis. Mice were treated with EVs + inhibitor NC, EVs + miR-182-5p inhibitor, or EVs + miR-182-5p inhibitor + si-CMTM7. **A** Tumor volume of nude mice, the statistical power was 1; **B** Tumor weight of nude mice after 28 days of modeling, the statistical power was 1; **C** Expression of miR-182-5p in tumor tissues of nude mice detected by RT-qPCR, the statistical power was 1; **D** Expression of CMTM7, p-EGFR, EGFR, VEGF, p-AKT and AKT protein in tumor tissues of nude mice detected by Western blot analysis, the statistical power was 1; **E**–**F** Number **E** and size (**F**) of pulmonary metastasis in nude mice, the statistical power was 1; **G**–**H** CD31 (**G**) and Ki-67 (**H**) immunohistochemical staining of pulmonary metastasis tissues in nude mice, the statistical power was 1. N = 10 for mice following each treatment. ****p < 0.0001 compared with mice treated with EVs + inhibitor NC. ####*p* < 0.0001 compared with HUVECs treated with EVs + miR-182-5p inhibitor + si-NC. The experiment was conducted three times independently

We constructed a nude mouse transplanted tumor to verify whether EVs-miR-182-5p can promote tumorigenesis and metastasis in vivo through the CMTM7/EGFR/AKT axis. As illustrated in Fig. 7A, B, the tumor volume and weight of mice treated with EVs + miR-182-5p inhibitor were reduced while they were increased in the presence of further CMTM7 silencing. The results of RT-qPCR and Western blot analysis (Fig. 7C, D) showed a decline of the expression of miR-182-5p in tumor tissues in response to treatment with the EVs + miR-182-5p inhibitor while the expression of CMTM7 was increased, along with decreased expression of VEGF and EGFR and AKT phosphorylation levels. There was no significant change in the expression of miR-182-5p in tumor tissues of mice treated with EVs + miR-182-5p inhibitor + si-CMTM7, while the expression of CMTM7 was reduced, and the EGFR and AKT phosphorylation levels were significantly increased.

In addition, the number and size of pulmonary metastasis were reduced in nude mice treated with EVs + miR-182-5p inhibitor but they were increased in the presence of EVs + miR-182-5p inhibitor + si-CMTM7 (Fig. 7E–F). CD31 and Ki-67 immunohistochemical staining results (Fig. 7G–H) showed reduced positive rate of CD31 and Ki-67 in pulmonary metastasis tissue of mice treated with EVs + miR-182-5p inhibitor but the positive rate was elevated in response to treatment with EVs + miR-182-5p inhibitor + si-CMTM7. The above results demonstrated that EVs-miR-182-5p targeted CMTM7 and activated the EGFR/AKT signaling pathway, thereby enhancing the tumorigenesis and metastasis of breast cancer cells in vivo.

## Discussion

Incidence and mortality in developed countries have been declining while increasing in developing countries, which may be due to changing risk factor profiles and difference in access to early detection and treatment of this cancer (Winters et al. 2017). The findings collected from this study supported that miR-182-5p delivered by breast cancer cell-derived EVs aggravated the development of breast cancer, which was related to its inhibition on the CMTM7 expression and activation on the EGFR/AKT signaling pathway. This may provide theoretical basis for potential treatment for breast cancer.

Initial results of the current study revealed that miR-182-5p was highly expressed in breast cancer tissues and cells, and this high expression was associated with poor prognosis of breast cancer patients. Meanwhile, miR-182-5p could promote the proliferation, migration and angiogenesis of HUVECs. Consistently, the expression of miR-182 has shown a dramatic overexpression in breast cancer tissue samples (Barsoum et al. 2020), and moreover, its overexpression significantly relates to poor clinical and pathological tumor characteristics in patients with locally advanced triple negative breast cancer and thus can be used as a tool to predict the outcomes and prognosis in these patients (Bajaj 2020). Downregulation of miR-182-5p contributes to inhibition of cell proliferation and promotion of tumor cell apoptosis by impairing the TLR4/NF-κB signaling pathway activity and increasing FBXW7 expression (Wu et al. 2020). VEGF is a growth factor with pro-angiogenic activity and can actively regulate the normal and pathological angiogenic processes due to its effects on increasing the vascular permeability (Melincovici 2018). In the conditioned medium of breast cancer cells overexpressing miR-182, VEGF-A expression is significantly upregulated and miR-182 can induce angiogenesis more efficiently in vitro (Chiang et al. 2016). Therefore, these findings provided an insight on the potential of miR-182-5p inhibition as a therapeutic target for the prevention and treatment of breast cancer.

Further findings suggested that breast cancer cell-derived EVs transferred miR-182-5p to HUVECs where it enhanced the migration, proliferation and angiogenesis. EVs released by cancer cells can transfer their bioactive cargoes such as mRNA, miRNA, and proteins to different recipient cells, thus facilitating cancer growth, immunosuppression and metastasis formation (Green et al. 2015). An increasing data have highlighted the promoting effect of tumor cell-derived EV cargo, especially the oncogenic miRNAs, on the cancer progression, such as angiogenesis, immune escape and tumor growth (Grange et al. 2019; Ludwig, et al. 2020; Shao et al. 2020), suggesting their potential as a therapeutic target for anti-angiogenic and anti-tumor therapies.

It has been well-established that miRNAs can interact with the 3’UTR of specific target mRNAs and consequently result in the inhibition of their expression (Ali Syeda et al. 2020). In this study, the biological prediction website and luciferase reporter assay identified that miR-182-5p bound to the 3’UTR of CMTM7 mRNA and could negatively regulate its expression. CMTM7 has been largely reported to be down-regulated and functions as a tumor suppressor by suppressing the cell proliferation, invasion and migration in vitro and tumorigenesis in vivo in a variety of cancers, including liver cancer and gastric cancer (Jin et al. 2018; Huang 2019). Moreover, CMTM7 is capable of increasing the internalization of EGFR, and further suppresses activation of the AKT signaling pathway during tumor pathogenesis (Li 2014). Activation of the EGFR/AKT signaling pathway correlates to the markedly enhanced migration and invasion of breast cancer cells (Liao 2021). Notably, the EGFR/AKT signaling pathway is strongly associated with tumor angiogenesis (Jin 2011). Considering the aforementioned data, we are convinced that EVs-miR-182-5p may promote breast cancer angiogenesis and the resultant cancer progression by blocking the CMTM7-mediated EGFR/AKT signaling pathway inactivation.

## Conclusions

To summarize all that we have gathered, the aforementioned data supported a conclusion that EVs-miR-182-5p may function as a driver of breast cancer progression and suggested a molecular mechanism that miR-182-5p bound to CMTM7 to activate the EGFR/AKT signaling pathway in this process (Additional file 4: Fig. S3). These novel findings uncovered in our study might become a cornerstone of the treatment for breast cancer in the future. Due to the limited data supporting the targeting between miR-182-5p and CMTM7, our subsequent endeavors are still required to fully realize the potential of this new mechanism.

## Supplementary Information

**Additional file 1: Table S1.** Basic information of datasets retrieved from the GEO database. **Table S2.** Primer sequences used for RT-qPCR

**Additional file 2: Fig. S1.** Identification of miR-182-5p in breast cancer by in situ hybridization staining. Representative staining results of miR-182-5p in adjacent normal (A) and tumor (B) tissues.

**Additional file 3: Fig. S2. **CMTM1 exerts suppressed HUVECs proliferation, migration and angiogenesis. HUVECs were treated with oe-CMTM7 or EVs or EVs + oe-CMTM7. The mRNA (A) and protein (B) expression of CMTM7 detected by RT-qPCR and Western blot analysis, respectively. The statistical power was 1; (C) The proliferation of HUVECs detected by CCK-8 assay, the statistical power was 1; (D) Migration ability of HUVECs detected by scratch test, the statistical power was 1; (E) Migration ability of HUVECs detected by Transwell assay, the statistical power was 1; (F) Vessel-like tube formation in vitro of HUVEC, the statistical power was 1. * *p* < 0.05, ** *p* < 0.01, *** *p* < 0.001, **** *p* < 0.0001 compared with HUVEC; # *p* < 0.05, ## *p* < 0.01, ### *p* < 0.001, #### *p* < 0.001compared with HUVEC treated with EVs. The experiment was conducted three times independently.

**Additional file 4: Fig. S3. **Molecular mechanism of EVs derived from tumor cells carrying miR-182-5p affect the breast cancer. Breast cancer cell-derived EVs transferred miR-182-5p to HUVECs, where it targeted CMTM7 and activated the EGFR/AKT signaling pathway, thus promoting the occurrence and development of breast cancer.

## Data Availability

All data generated or analyzed during this study are included in this published article.

## Abbreviations

EVs: Extracellular vesicles
miRNAs: MicroRNAs
CMTM7: CKLF-like MARVEL transmembrane domain-containing 7
HUVECs: Human umbilical vein endothelial cells
EGFR/AKT: Epidermal growth factor receptor/protein kinase B (EGFR/AKT)
KEGG: Kyoto encyclopedia of genes and genomes
WHO: World Health Organization
TNM: Tumor-lymph node-metastasis
ISH: In situ hybridization
DLS: Dynamic light scattering
TEM: Transmission electron microscope
PTFE: Polytetrafluoroethylene
PC: Polycarbonate
ANOVA: Analysis of variance
