# Supplementary material for: Breast cancer cell-derived extracellular vesicles transfer miR-182-5p and promote breast carcinogenesis via the CMTM7/EGFR/AKT axis
Source: Mol Med. 2021 Jul 16;27:78. doi: 10.1186/s10020-021-00338-8 (PMC8296627; doi:10.1186/s10020-021-00338-8)
Supplement: Supplementary file 1 — Additional file 1: Table S1. Basic information of datasets retrieved from the GEO database. Table S2. Primer sequences used for RT-qPCR [file 10020_2021_338_MOESM1_ESM.docx]

**Table S1** Basic information of datasets retrieved from the GEO database

| GSE# ID | Platforms | Samples | Normal | Tumor | Description |
| --- | --- | --- | --- | --- | --- |
| GSE58027 | GPL15446 | 4 | 2 | 2 | Delete 4 other samples |
| GSE45666 | GPL14767 | 116 | 15 | 101 | log2 processing of data is required |
| GSE35412-1 | GPL9731 | 40 | 6 | 34 |  |
| GSE35412-2 | GPL9732 | 35 | 6 | 29 |  |
| GSE26659 | GPL8227 | 94 | 17 | 77 |  |
| GSE33447 | GPL14550 | 16 | 8 | 8 | log2 processing of data is required |
| GSE3744 | GPL570 | 47 | 7 | 40 |  |
| GSE50428 | GPL13648 | 31 | 5 | 26 |  |

**Table S2** Primer sequences used for RT-qPCR

| RNA | Primer sequence |
| --- | --- |
| miR-182-5p | Forward: 5’-GGCAATGGTAGAACTCAC-3’ |
|  | Reverse: 5’-GAACATGTCTGCGTATCTC-3’ |
| miR-183 | Forward: 5’-ATGGCACTGGTAGAATTC-3’ |
|  | Reverse: 5’-GAACATGTCTGCGTATCTC-3’´ |
| miR-454 | Forward: 5’-CCTATCAATATTGTCTCTG-3’ |
|  | Reverse: 5’-GAACATGTCTGCGTATCTC-3’ |
| miR-425 | Forward: 5’-ATGACACGATCACTCCC-3’ |
|  | Reverse: 5’-GAACATGTCTGCGTATCTC-3’ |
| miR-145 | Forward: 5’-GTCCAGTTTTCCCAGGA-3’ |
|  | Reverse: 5’-GAACATGTCTGCGTATCTC-3’ |
| miR-130b | Forward: 5’-CTCTTTCCCTGTTGCAC-3’ |
|  | Reverse: 5’-GAACATGTCTGCGTATCTC-3’ |
| miR-99a | Forward: 5’-AACCCGTAGATCCGATC-3’ |
|  | Reverse: 5’-GAACATGTCTGCGTATCTC-3’ |
| miR-143 | Forward: 5’-GCAGTGCTGCATCTCTG-3’ |
|  | Reverse: 5’-GAACATGTCTGCGTATCTC-3’ |
| CMTM7 | Forward: 5’-CCTGCTGAAAGTGGCGCAAATG-3’ |
|  | Reverse: 5’-TGGACCAGGTAAAAGGCGAGGA-3’ |
| U6 | Forward: 5’-CTCGCTTCGGCAGCACA-3’ |
|  | Reverse: 5’-AACGCTTCACGAATTTGCGT-3’ |
| GAPDH | Forward: 5’-GGGCCAAAAGGGTCATCATC-3’ |
|  | Reverse: 5’-ATGACCTTGCCCACAGCCTT-3’ |
