# Supplementary figures and images for: Breast cancer cell-derived extracellular vesicles transfer miR-182-5p and promote breast carcinogenesis via the CMTM7/EGFR/AKT axis
Source: Mol Med. 2021 Jul 16;27:78. doi: 10.1186/s10020-021-00338-8 (PMC8296627; doi:10.1186/s10020-021-00338-8)

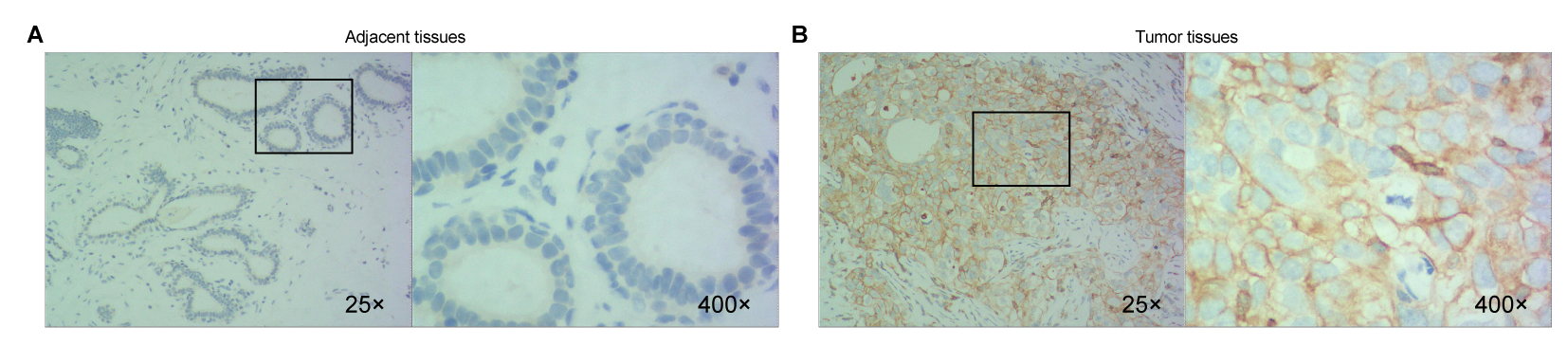

Supplement: Supplementary file 2 — Additional file 2: Fig. S1. Identification of miR-182-5p in breast cancer by in situ hybridization staining. Representative staining results of miR-182-5p in adjacent normal (A) and tumor (B) tissues. [file 10020_2021_338_MOESM2_ESM.jpg]

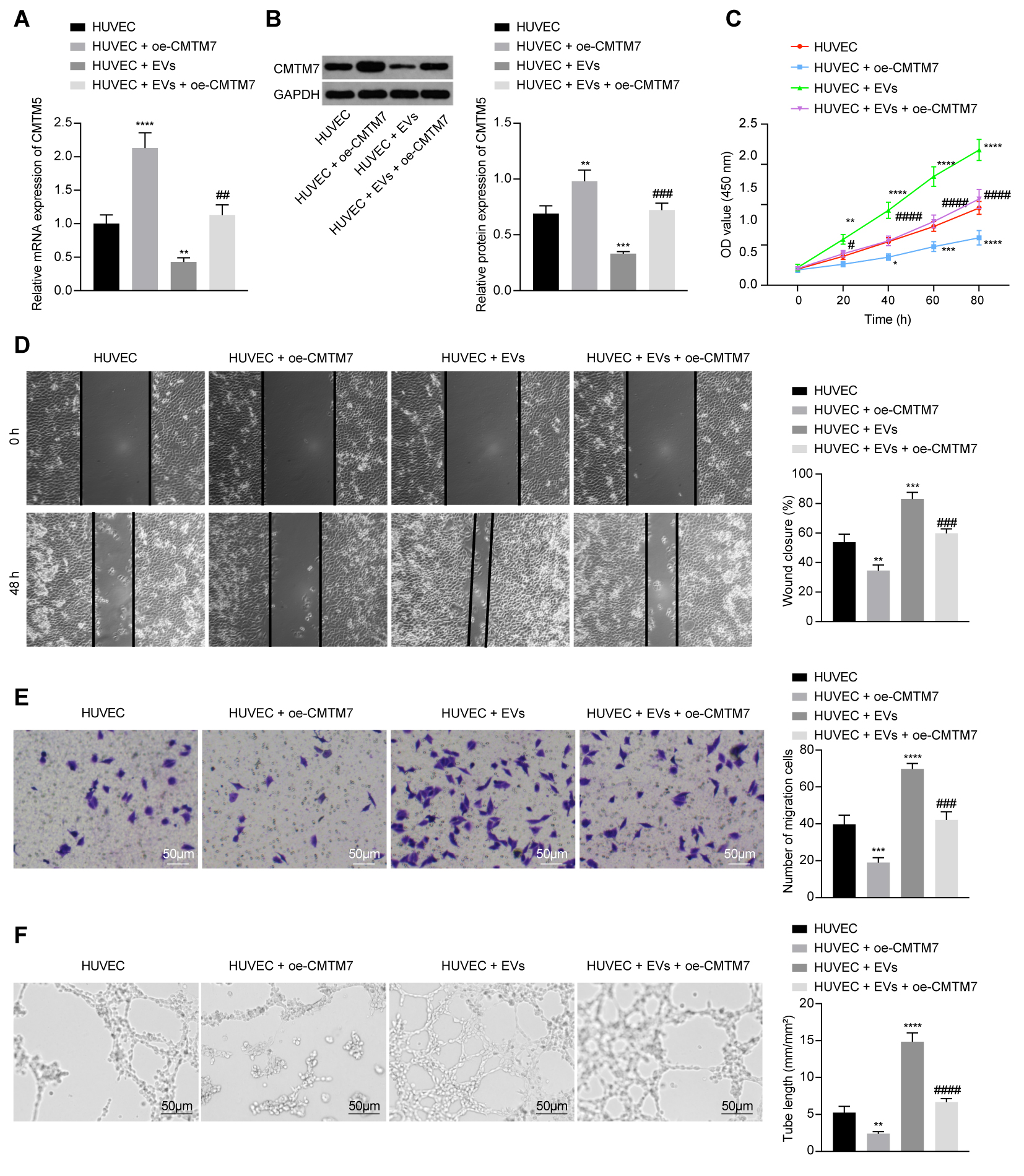

Supplement: Supplementary file 3 — Additional file 3: Fig. S2. CMTM1 exerts suppressed HUVECs proliferation, migration and angiogenesis. HUVECs were treated with oe-CMTM7 or EVs or EVs + oe-CMTM7. The mRNA (A) and protein (B) expression of CMTM7 detected by RT-qPCR and Western blot analysis, respectively. The statistical power was 1; (C) The proliferation of HUVECs detected by CCK-8 assay, the statistical power was 1; (D) Migration ability of HUVECs detected by scratch test, the statistical power was 1; (E) Migration ability of HUVECs detected by Transwell assay, the statistical power was 1; (F) Vessel-like tube formation in vitro of HUVEC, the statistical power was 1. * p < 0.05, ** p < 0.01, *** p < 0.001, **** p < 0.0001 compared with HUVEC; # p < 0.05, ## p < 0.01, ### p < 0.001, #### p < 0.001compared with HUVEC treated with EVs. The experiment was conducted three times independently. [file 10020_2021_338_MOESM3_ESM.jpg]

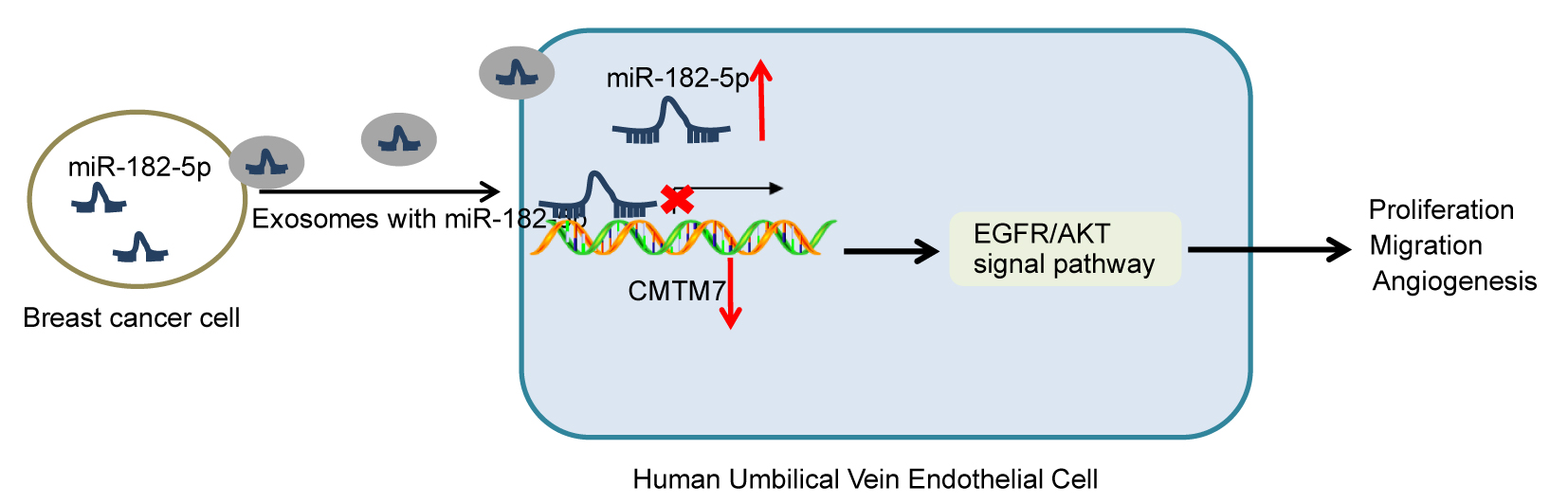

Supplement: Supplementary file 4 — Additional file 4: Fig. S3. Molecular mechanism of EVs derived from tumor cells carrying miR-182-5p affect the breast cancer. Breast cancer cell-derived EVs transferred miR-182-5p to HUVECs, where it targeted CMTM7 and activated the EGFR/AKT signaling pathway, thus promoting the occurrence and development of breast cancer. [file 10020_2021_338_MOESM4_ESM.jpg]
